# A genomic association study revealing subphenotypes of childhood steroid-sensitive nephrotic syndrome in a larger genomic sequencing cohort

**DOI:** 10.1016/j.gendis.2023.101126

**Published:** 2023-09-27

**Authors:** Han Chan, Fenfen Ni, Bo Zhao, Huimin Jiang, Juanjuan Ding, Li Wang, Xiaowen Wang, Jingjing Cui, Shipin Feng, Xiaojie Gao, Xueying Yang, Huan Chi, Hao Lee, Xuelan Chen, Xiaoqin Li, Jia Jiao, Daoqi Wu, Gaofu Zhang, Mo Wang, Yupeng Cun, Xiongzhong Ruan, Haiping Yang, Qiu Li

**Affiliations:** a Department of Nephrology, Children's Hospital of Chongqing Medical University, National Clinical Research Center for Child Health and Disorders, Ministry of Education Key Laboratory of Child Development and Disorders, Chongqing Key Laboratory of Pediatrics, Chongqing 400014, China; b Department of Nephrology, Shenzhen Children's Hospital, Shenzhen, Guangdong 518034, China; c Department of Nephrology, Kunming Children's Hospital, Kunming Medical University, Kunming, Yunnan 650228, China; d Department of Nephrology, Wuhan Children's Hospital, Tongji Medical College of Huazhong University of Science and Technology, Wuhan, Hubei 430015, China; e Department of Nephrology, Chengdu Women and Children Central Hospital, Chengdu, Sichuan 610073, China; f Pediatric Research Institute, Ministry of Education Key Laboratory of Child Development and Disorders, National Clinical Research Center for Child Health and Disorders, China International Science and Technology Cooperation Base of Child Development and Critical Disorders, Chongqing Key Laboratory of Translational Medical Research in Cognitive Development and Learning and Memory Disorders, Children's Hospital of Chongqing Medical University, Chongqing 400014, China; g Department of Nephrology, John Moorhead Research Laboratory, University College London Medical School, Royal Free Campus, University College London, London NW3 2PF, United Kingdom

**Keywords:** Frequent relapse, Genome-wide association study, Human leukocyte antigen region, Steroid-sensitive nephrotic syndrome

## Abstract

Dissecting the genetic components that contribute to the two main subphenotypes of steroid-sensitive nephrotic syndrome (SSNS) using genome-wide association studies (GWAS) strategy is important for understanding the disease. We conducted a multicenter cohort study (360 patients and 1835 controls) combined with a GWAS strategy to identify susceptibility variants associated with the following two subphenotypes of SSNS: steroid-sensitive nephrotic syndrome without relapse (SSNSWR, 181 patients) and steroid-dependent/frequent relapse nephrotic syndrome (SDNS/FRNS, 179 patients). The distribution of two single-nucleotide polymorphisms (SNPs) in *ANKRD36* and *ALPG* was significant between SSNSWR and healthy controls, and that of two SNPs in *GAD1* and *HLA-DQA1* was significant between SDNS/FRNS and healthy controls. Interestingly, rs1047989 in *HLA-DQA1* was a candidate locus for SDNS/FRNS but not for SSNSWR. No significant SNPs were observed between SSNSWR and SDNS/FRNS. Meanwhile, chromosome 2:171713702 in *GAD1* was associated with a greater steroid dose (>0.75 mg/kg/d) upon relapse to first remission in patients with SDNS/FRNS (odds ratio = 3.14; 95% confidence interval, 0.97–9.87; *P* = 0.034). rs117014418 in *APOL4* was significantly associated with a decrease in eGFR of greater than 20% compared with the baseline in SDNS/FRNS patients (*P* = 0.0001). Protein–protein intersection network construction suggested that HLA-DQA1 and HLA-DQB1 function together through GSDMA. Thus, SSNSWR belongs to non-HLA region-dependent nephropathy, and the HLA-DQA/DQB region is likely strongly associated with disease relapse, especially in SDNS/FRNS. The study provides a novel approach for the GWAS strategy of SSNS and contributes to our understanding of the pathological mechanisms of SSNSWR and SDNS/FRNS.

## Introduction

Idiopathic nephrotic syndrome is the most common cause of chronic glomerular disease in children. During initial treatment, 77.6%–90.0% of idiopathic nephrotic syndrome cases are glucocorticoid sensitive (steroid-sensitive nephrotic syndrome, SSNS).^1^^,^^2^ SSNS can be divided into three subphenotypes based on the treatment response in conventional clinical practice: steroid-sensitive nephrotic syndrome without relapse (SSNSWR), steroid-sensitive nephrotic syndrome without frequent relapse, and steroid-dependent nephrotic syndrome/frequent relapse nephrotic syndrome (SDNS/FRNS).^3^^,^^4^ In particular, approximately half of SSNS patients who suffer relapse have a higher risk than other subsets for developing FRNS.

SSNS is generally considered an autoimmune disease, and its etiopathogenesis remains unknown, especially regarding the genetic mechanism of SSNSWR and SDNS/FRNS. The former is characterized by no relapse after the start of steroid therapy; the latter is characterized by repetitive relapse and remission, although no patient has a known genetic mutation or positive family history of nephrotic syndrome. Therefore, the internal mechanism between SSNSWR and SDNS/FRNS underlying different responses to steroid therapy remains unclear. According to current studies, most SSNS cases are likely due to genetic variants that cause structural and functional defects in glomerular visceral epithelial cells, and the clinical and pathological phenotypes of each subgroup are potentially tightly linked to the genetic phenotype.^2^^,^^5^^,^^6^ The genome-wide association study (GWAS) strategy is an efficient method for detecting genetic risk loci in complex diseases. As a result of the difficulty in distinguishing different types of reactions to steroid onset in clinical practice, the current strategy of GWAS focuses mainly on identifying risk loci associated with SSNS. Most associations identified by GWAS in SSNS, which is the most common form of idiopathic nephrotic syndrome, are in the human leukocyte antigen (*HLA*) region, which is associated with most autoimmune or infectious diseases. In 2014, Gbadegesin et al^7^ first conducted an exome array study and identified rs1129740 and rs1071630, which are located within *HLA-DQA1*, as candidate loci for SSNS in South Asian and White European patients. Adeyemo et al^8^ further confirmed that *HLA-DQA1* is a risk locus for SSNS in African–American patients, which is consistent with its role in determining SSNS risk in children of European, Asian, and African ancestry. In 2018, Jia et al^9^ performed a GWAS with a replication study in a Japanese population and showed that the *HLA-DR/DQ* region is associated with childhood SSNS. Furthermore, a transethnic meta-analysis identified rs1063348 as related to decreased glomerular expression of *HLA-DRB1*, *HLA-DRB5*, and *HLA-DQB1*, potentially leading to immune dysregulation in SSNS.^10^^,^^11^ Despite all these findings, published studies have confirmed that the HLA region is associated with SSNS. However, due to the limited number of SSNSWR and SDNS/FRNS patients, the influence of the HLA region on the disease process of SSNS remains unclear.

China has a large and diverse population of SSNS patients, which enables the recruitment of SSNSWR and SDNS/FRNS patients for larger cohort genomic analyses. In this study, we collected a larger cohort of SSNSWR and SDNS/FRNS patients for whole-exome sequencing from multiple collaborating centers in China, performed genomic analysis, and applied a GWAS strategy to identify risk variants associated with SSNS subphenotypes. Then, a clinical replication was conducted to study how these variants influence clinical phenotype and treatment outcome. This study will enhance our understanding of how these genetic variant factors contribute to the treatment and prognosis management of idiopathic nephrotic syndrome.

## Material and methods

### Patient cohort

A retrospective cohort study was performed at the following five collaborating hospitals in China: Children's Hospital of Chongqing Medical University, West China Women's and Children's Hospital, Shenzhen Children's Hospital, Wuhan Children's Hospital, and Kunming Children's Hospital. The included patients with SSNSWR or SDNS/FRNS aged three months to eighteen years were recruited between March 2019 and June 2022. They had complete retrospective medical records at each respective center from the beginning of the disease course. The detailed inclusion and exclusion criteria are presented in Table S15. Genomic DNA was extracted from peripheral blood using a standard protocol, and whole-exome sequencing was conducted when a case progressed to a confirmed clinical phenotype (SSNSWR and SDNS/FRNS) during follow-up. The study was approved by the Ethics Committee of the China National Clinical Research Centre (Children's Health and Disease, reference number 2019-34-1).

The recruitment phase included 181 SSNSWR and 179 SDNS/FRNS patients of Chinese Han ethnicity. Among these patients, 214 (59.44%) were recruited from the Children's Hospital of Chongqing Medical University (106 SSNSWR and 108 SDNS/FRNS), 73 (20.28%) from West China Women's and Children's Hospital (45 SSNSWR and 28 SDNS/FRNS), 44 (12.22%) from Shenzhen Children's Hospital (18 SSNSWR and 26 SDNS/FRNS), 18 (5%) from Wuhan Children's Hospital (8 SSNSWR and 10 SDNS/FRNS), and 11 (3.06%) from Kunming Children's Hospital (4 SSNSWR and 7 SDNS/FRNS). The remaining 1835 healthy controls consisting of adults and children used in the GWAS analysis were recruited from the MyGenostics database and underwent genotyping with the GenCap whole-exome sequencing capture kit (MyGenostics, Beijing) and the Illumina NovaSeq platform, with a unified analysis process and quality control conditions.

### Genotyping and quality control for whole-exome sequencing data

Genotyping and quality control were performed using standard procedures,^12^, ^13^, ^14^ and the details are listed in Supplementary Methods.

### GWAS strategy and conditional analysis

A GWAS strategy of SSNSWR and SDNS/FRNS patients was performed, and each was compared with healthy controls using PLINK 2.0 by using an additive multivariable logistic regression model across the whole-exome sequencing data.^15^ To control the unbalanced case‒control ratios and sample relatedness, the Scalable and Accurate Implementation of the GEneralized (SAIGE) mixed model was also used for the top SNPs.^16^ Covariates, including basic variables, such as age, sex, and genetic PCs, were used in the regression to adjust for any sources of clinical trait variability. Haplotype block analysis was conducted to determine the range of candidate regions by LDBlockShow 1.4.^17^ A genome-wide significance threshold of *P* < 5.0 × 10^−8^ was used.

### Gene-based analysis

Given that the study of coding variation using the GenCap whole-exome sequencing capture kit gives rise to both common and rare variants, we performed gene set rare variant (minor allele frequency < 1%) analysis in the patient cohort using the sequence kernel association test-O (SKAT-O) as implemented in the Efficient and Parallelizable Association Container Toolbox (EPACTS) software (Hyun Min Kang, Ann Arbor, MI, USA) (https://genome.sph.umich.edu/wiki/EPACTS), which can easily be applied to genome-wide or exome-wide data.

### Annotation and structural modeling of variants

Swiss-model, I-TASSAR, and trRosetta were used to predict changes in protein structure.^18^, ^19^, ^20^, ^21^

### Clinical replication and follow-up

This study followed the Improving Global Outcomes Glomerulonephritis Work Group (KDIGO) guidelines for the use of immunosuppressants and evidence-based practices to explain treatment options to the parents of patients and make decisions regarding the use of immunosuppressants.^22^ The patients were evaluated from study entry to the time of the latest follow-up. The follow-up data for SSNSWR and SDNS/FRNS patients will be collected and used to identify the risk variants that potentially influence the clinical manifestations and treatment outcomes. A *P* value <5.0 × 10^−7^ was considered the threshold for clinical replication.

### Statistical analyses

For multiple comparisons of genetic variants, all *P* values were corrected with the Bonferroni method. The false discovery rate-adjusted significance was used to control for multiple comparisons between SSNSWR and SDNS/FRNS, and corrected *P* values below 0.05 were considered statistically significant. Kaplan–Meier analysis was employed to analyze factors influencing treatment outcomes in follow-up and decreasing the estimated glomerular filtration rate (eGFR).

## Results

**Figure 1:**
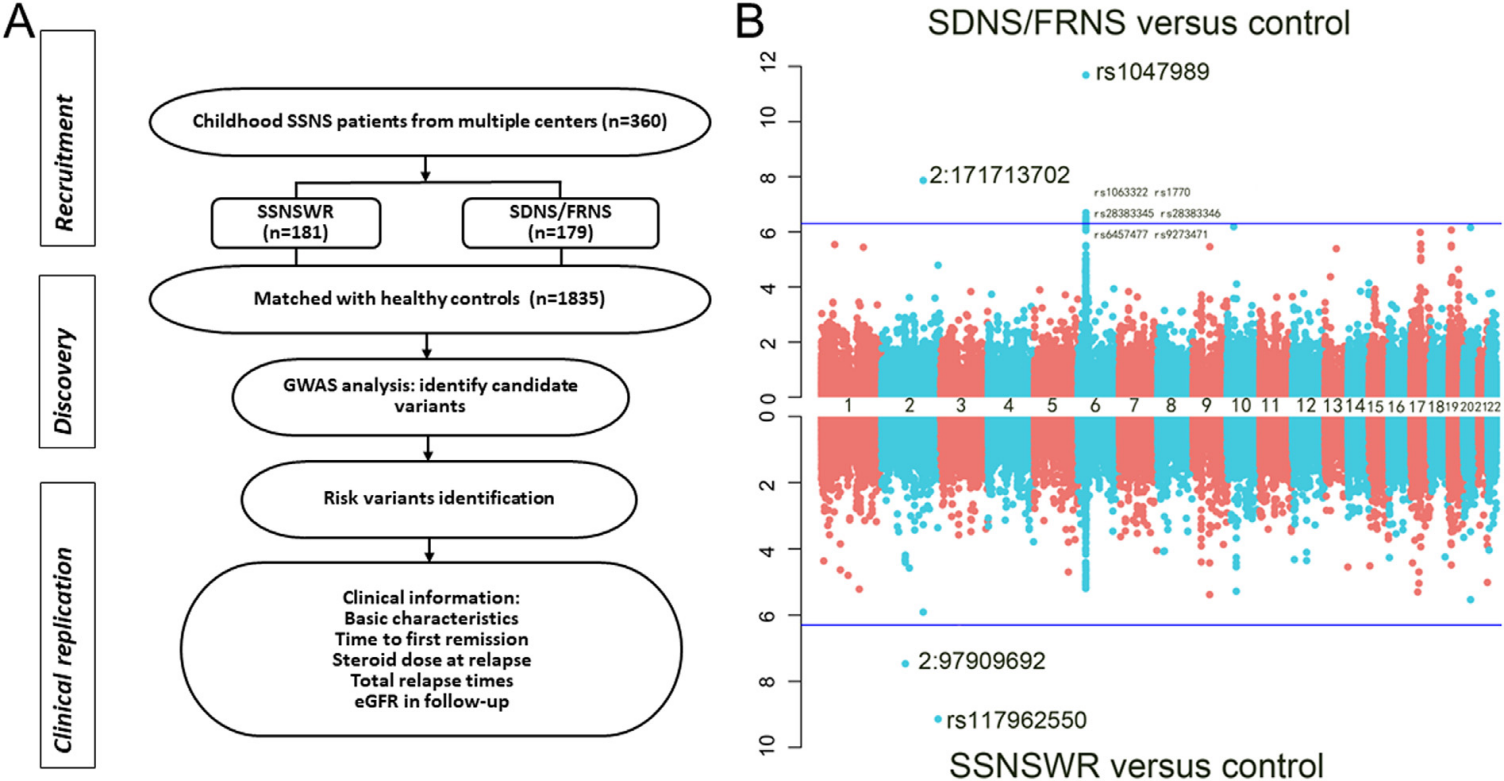
Main outline of the discovery cohort. **(A)** Flowchart of the cohort study design. **(B)** Manhattan plot of single-marker association *P* values. Observed *P* values are plotted on the y-axis, and chromosome locations are plotted on the x-axis. The red line indicates the genome-wide significance threshold (5.0 × 10^−8^). The blue line indicates the threshold of *P* value <5.0 × 10^−7^.

This study involved 360 patients with idiopathic nephrotic syndrome, including 181 with SSNSWR and 179 with SDNS/FRNS, of Chinese Han ethnicity from five hospitals in China (Fig. 1A). The characteristics of the patients in the two groups at registration are provided in Table 1. The patient and control samples clustered closely together. The plots of principal component 1 versus principal component 2 showed that the patient samples clustered into Southern Han Chinese ancestry and separately from populations of European, American, Japanese, and African ancestry (Fig. S1, 2). After stringent quality control and exclusion of sex chromosome SNPs, 76,423 SNPs remained for the 174 SSNSWR and 170 SDNS/FRNS cases (Table S2, 3). The plot of linkage disequilibrium decay suggested no significant differences among the three groups, namely, the two above groups and controls (Fig. S3). There was no sex discrepancy in the study, and the sample sex was consistent with the clinical information.

**Table 1:** Summary of the clinical details.

Characteristics	SSNSWR (*n* = 181)	SDNS/FRNS (*n* = 179)	*P*
Sex [*n* (%)]			
Male	137 (75.69)	132 (73.74)	0.671
Female	44 (24.31)	47 (26.26)	
Age onset (month)	53.16 ± 33.80	53.23 ± 35.86	0.984
Body mass index (kg/m^2^)	18.05 ± 3.13	18.28 ± 3.17	0.316
Family history [*n* (%)]			0.067
No	176 (97.24)	172 (96.09)	
Yes	4 (2.21)	1 (0.56)	
Unknown	1 (0.55)	6 (3.35)	
Time to first remission (days)	9.81 ± 4.80	12.80 ± 6.21	**<0.001**
eGFR onset (mL/min·1.73 m^2^)	139.37 ± 44.29	135.26 ± 34.87	0.375
Follow-up time (month)	37.24 ± 17.20	47.44 ± 30.38	**0.000**
12–24 or equal to 24 [*n* (%)]	45 (24.86)	42 (23.46)	
24–36 or equal to 36 [*n* (%)]	53 (29.28)	38 (21.23)	
More than 36 [n (%)]	83 (45.86)	99 (55.30)	
Histology (first completed) [*n* (%)]			
Minimal change disease	5 (2.76)	73 (40.78)	
Focal segmental glomerular sclerosis	0 (0.00)	2 (1.12)	
Mesangial proliferative glomerulonephritis	0 (0.00)	4 (2.23)	
No biopsy	176 (97.24)	100 (55.87)	
Last follow-up status [*n* (%)]			
No remission	0 (00.00)	2 (1.12)	
Partial remission	0 (00.00)	60 (33.52)	
Complete remission	27 (14.91)	100 (55.87)	
Clinically cured	151 (83.43)	3 (1.68)	
Unable to determine	3 (1.66)	14 (7.82)	

Notes: SSNSWR, steroid-sensitive nephrotic syndrome without relapse; SDNS/FRNS, steroid-dependent/frequent relapse nephrotic syndrome; eGFR, estimated glomerular filtration rate; eGFR onset, estimated glomerular filtration rate by updated Schwartz formula at study entry; Time to first remission, the date from the initial steroid therapy onset when an idiopathic nephrotic syndrome patient's morning urine protein is negative; Unable to determine, missing data. *P* values lower than 0.05 are shown in bold.

### Results of the GWAS strategy

#### SSNSWR versus control

**Figure 2:**
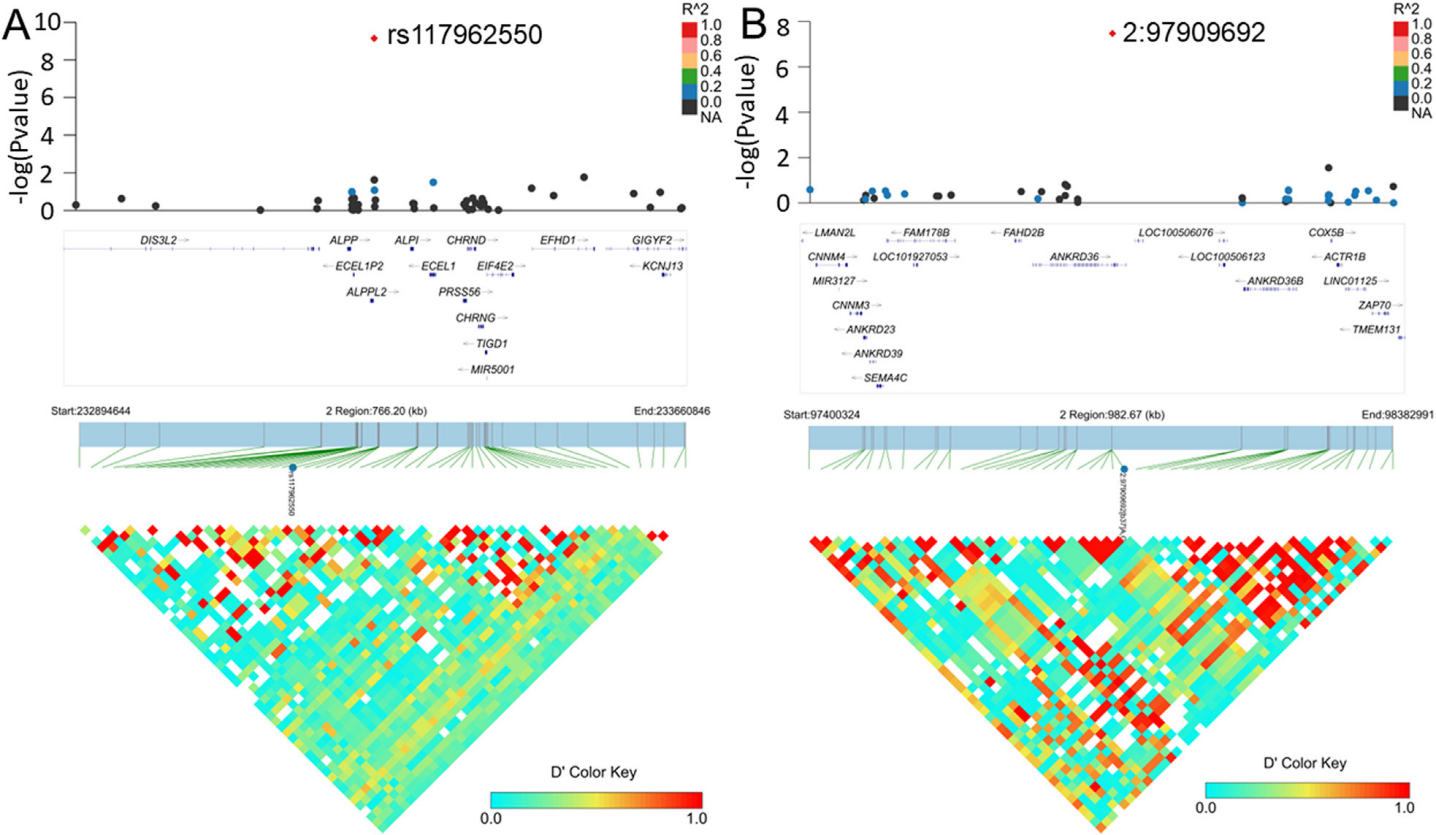
The regional plot of the chromosome 2 region showing rs117962550 and 2:97909692 to be independent of one haplotype.

The GWAS strategy revealed the genome-wide significance of one chromosome (Fig. 1B). The quantile–quantile plots are presented in Figure S4A. Interestingly, no significant SNPs in the *HLA* region were detected between the SSNSWR and control groups. Two SNPs are located on chromosome 2 (rs117962550, odds ratio/OR = 4.13, 95% confidence interval/CI: 2.63–6.49, *P* = 7.17 × 10^−10^; 2:97909692, OR = 2.62, 95% CI: 1.86–3.68, *P* = 3.37 × 10^−8^), and conditioning for these two SNPs abolished all evidence of an association with genome-wide significance (Fig. 2). SAIGE test with Bonferroni adjustment further identified rs117962550 as an independent variant strongly associated with SSNSWR (*P* = 4.15 × 10^−8^). The most significant signal in the *ALPG* region was at 2q37.1 (rs117962550), and the details of the major genotyped SNPs at the respective loci are listed in Table 2.

**Table 2:** Details of the main candidate single-nucleotide polymorphisms.

Chromosome	SNP	BP	A1	OR	L95	U95	*P*	Subgroup [MAF]	Control group [MAF]	Location	Gene
*SSNSWR vs. control*											
chr2	rs117962550	233271731	A	4.134	2.632	6.492	7.17E-10	36/174 [0.103]	126/1798 [0.035]	Intronic	*ALPG*
chr2	2:97909692	97909692	A	2.617	1.86	3.682	3.37E-08	67/174 [0.195]	317/1793 [0.090]	Exonic	*ANKRD36*
chr1	rs746236012	173569393	A	4.395	2.548	7.58	1.02E-07	24/174 [0.069]	75/1791 [0.021]	Intronic	*SLC9C2*
*SDNS/FRNS vs. control*											
chr6	rs1047989	32605257	A	0.389	0.299	0.506	2.08E-12	87/170 [0.315]	1299/1798 [0.471]	Exonic	*HLA-DQA1*
chr2	2:171713702	171713702	T	5.704	3.127	10.4	1.37E-08	21/170 [0.062]	46/1773 [0.013]	Intronic	*GAD1*
chr19	rs774409792	8997499	A	4.147	2.444	7.035	1.34E-07	25/170 [0.074]	82/1798 [0.023]	Exonic	*MUC16*
chr22	rs117014418	36587404	A	3.122	2.04	4.777	1.59E-07	33/170 [0.103]	143/1798 [0.041]	Exonic	*APOL4*
chr6	rs1063322	32629935	G	0.496	0.379	0.650	3.39E-07	78/170 [0.277]	1113/1798 [0.384]	Exonic	*HLA-DQB1*
chr6	rs1770	32627833	G	1.924	1.503	2.463	2.03E-07	133/169 [0.512]	1152/1794 [0.401]	ncRNA	*HLA-DQB1*
chr6	rs28383345	32605234	A	3.246	2.071	5.087	2.80E-07	32/170 [0.094]	313/1798 [0.091]	UTR5	*HLA-DQA1*
chr6	rs28383346	32605326	G	3.225	2.058	5.054	3.21E-07	32/170 [0.094]	314/1798 [0.091]	Splicing	*HLA-DQA1*
chr6	rs6457477	31977391	T	2.148	1.598	2.888	4.12E-07	74/169 [0.243]	689/1773 [0.212]	Exonic	*TNXB*
chr6	rs9273471	32628030	A	1.899	1.481	2.434	4.17E-07	131/169 [0.497]	1139/1796 [0.393]	Splicing	*HLA-DQB1*
chr9	rs139880713	68433568	C	4.794	2.646	8.685	2.36E-07	20/170 [0.059]	55/1798 [0.015]	ncRNA	*FRG1JP*

Notes: *P* values lower than 5.0 × 10^−8^ are shown in bold. SNP, single-nucleotide polymorphism; BP, physical position (base-pairs); A1, minor allele name (based on whole sample); OR, estimated odds ratio for A1; L95, lower bound of 95% confidence interval for odds ratio; U95, upper bound of 95% confidence interval for odds ratio; *P*, asymptotic *P*-value for this test; MAF, minor allele frequency; SSNSWR, steroid-sensitive nephrotic syndrome without relapse; SDNS/FRNS, steroid-dependent/frequent relapse nephrotic syndrome.

#### SDNS/FRNS versus control

**Figure 3:**
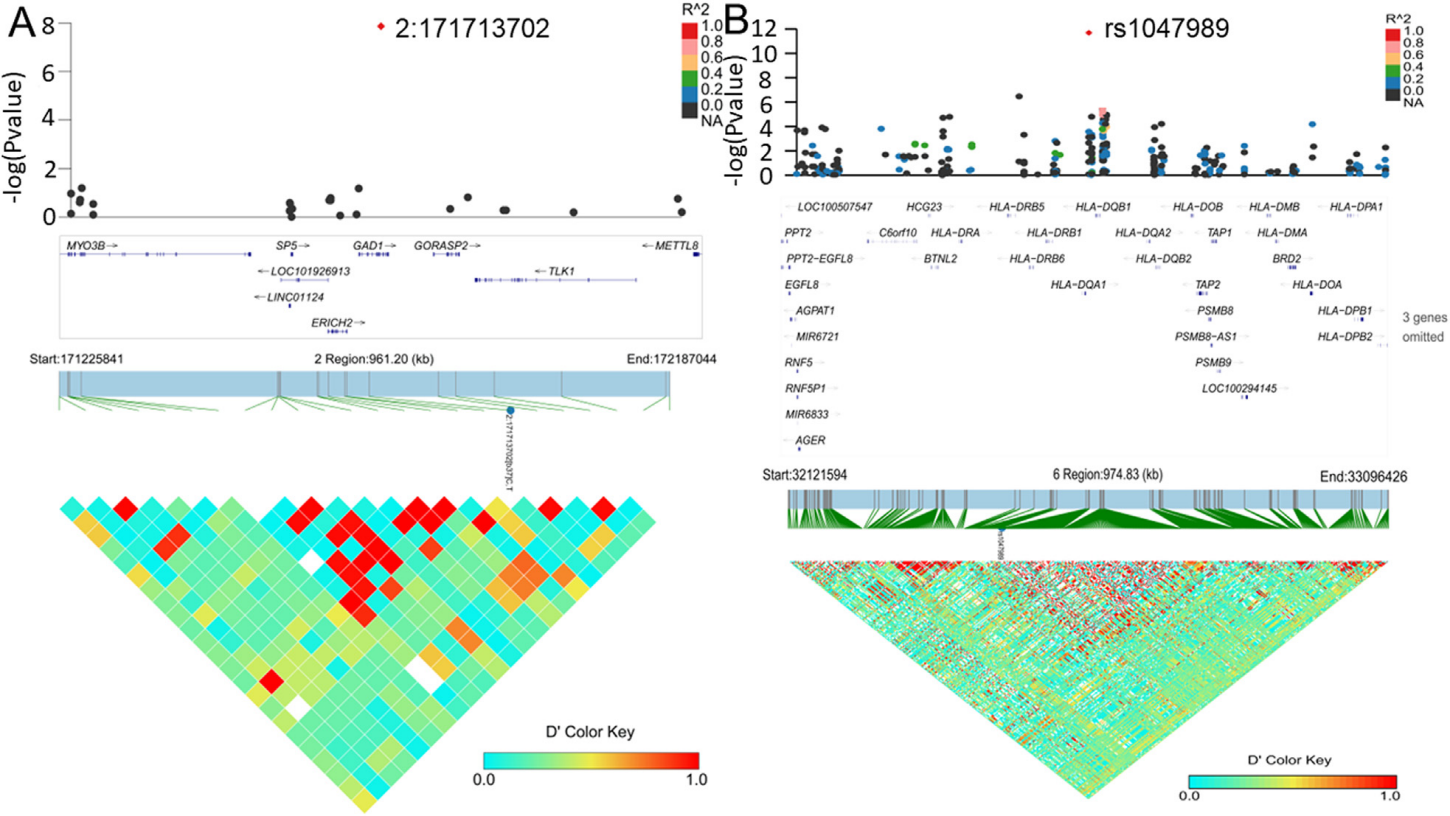
The regional plot of the chromosome 6 region showing 2:171713702 and rs1047989 to be independent of one haplotype.

Two chromosomes showed genome-wide significance (Fig. 1B). The quantile–quantile plots are presented in Figure S4B. One SNP is located on chromosome 2 (2:171713702), and one SNP is located on chromosome 6 (rs1047989). Notably, except for rs6457477 in *TNXB*, there were clustered SNPs (rs1063322, rs1770, rs28383345, rs28383346, rs6457477, and rs9273471) on chromosome 6 in the *HLA-DQA1*, *HLA-DQB1*, and *HLA-DRB5* regions that were significantly associated with SDNS/FRNS under the threshold of a *P* value <5.0 × 10^−7^ (Table 2), suggesting that the *HLA* region plays important roles in the development of SDNS/FRNS. The SAIGE test showed that the variant with the strongest evidence for these associations was rs1047989 (*P* = 5.14 × 10^−12^). Conditioning for the top SNPs on each chromosome (2:171713702, rs1047989) abolished all evidence of an association with genome-wide significance (Fig. 3). The details of the main genotyped SNPs at the respective loci are listed in Table 2.

#### SSNSWR versus SDNS/FRNS

Both the genome-wide significance threshold (*P* < 5.0 × 10^−8^) and false discovery rate-adjusted significance were determined. However, no significant SNPs were observed between the SSNSWR and SDNS/FRNS groups. The top two signals in the *GSDMA* region were at 17q21.1 (rs7212938, OR = 0.50, 95% CI: 0.36–0.69, *P* = 2.73 × 10^−5^; rs9914973, OR = 0.49, 95% CI: 0.36–0.69, *P* = 3.75 × 10^−5^). The SAIGE results reaffirmed this conclusion (rs7212938, *P* = 2.10 × 10^−5^; rs9914973, *P* = 3.13 × 10^−5^). The quantile–quantile plots are depicted in Figure S4C. The details of the top 10 genotyped SNPs at the respective loci are listed in Table S4.

### Expression quantitative trait locus (eQTL) analysis

To explore the regulatory mechanism of the above variants, we combined eQTL and GWAS strategy results to further screen candidate genes that might contribute to SSNS. The GTEx database did not reveal any significant eQTLs for rs117962550, 2:97909692, 2:171713702, and rs1047989 in any tissue.^23^^,^^24^ In the NephQTL browser, one significant SNP (rs1047989) had strong cis-eQTL effects that also involved the neighboring genes *HLA-DQB1*, *HLA-DRB5*, *HLA-DRB1*, *HLA-DQB2*, *HLA-DQA2*, *PSMB9*, *HCG23*, *PSMB8-AS1*, and *HLA-DPB2*. In contrast, no significant eQTLs for rs117962550, 2:97909692, and 2:171713702 were found in any tissue in the NephQTL database. Ranking normalized, adjusted glomerular expression for rs1047989 is listed in Figure S5.

### Gene-based analysis for SSNSWR and SDNS/FRNS

To identify significant genes with multiple causal variants, we conducted a gene-based SKAT-O test on rare variants. In the comparison between the SSNS and control groups, the most strongly associated gene set was *CPNE4* (*P* = 1.28 × 10^−30^, SSNSWR *vs*. control; *P* = 1.32 × 10^−10^, SDNS/FRNS *vs*. control) at a rare-variant minor allele frequency threshold of 0.01. According to the UniProt Consortium,^25^ *CPNE4* encodes a calcium-dependent, phospholipid-binding protein that may be involved in calcium-mediated intracellular processes. However, no significant SNPs were observed between the SSNSWR and SDNS/FRNS groups. This finding was highly consistent with the single-marker common-variant association (Table S6).

### Clinical replication

#### Genetic pattern analysis

All observed significant SNPs were analyzed via genetic models (recessive, dominant, and additive models). The additive model provided the best fit for rs117962550. The dominant model provided the best fit for rs746236012, 2:97909692, 2:171713702, rs1047989, rs139880713, rs774409792, and rs117014418 (Table S7–14). These results suggest a trend of increased risk for patients in the heterozygous and homozygous states.

#### Time to first remission

The clinical details of the SDNS/FRNS cases evaluated are presented in Table 3. No association was observed between potential significant SNPs and time to first remission after patients with idiopathic nephrotic syndrome (SSNSWR and SDNS/FRNS) received steroid treatment.

**Table 3:** Treatment characteristics of SDNS/FRNS patients.

Characteristics	SDNS/FRNS (*n* = 179) (%)
Total relapse times (*n*)	3.30 ± 2.00
2–3 times	121 (67.60)
More than 3 times	52 (29.05)
Unable to determine	6 (3.35)
Average relapse frequency (*n*/follow-up years)	
Less than or equal to 1∗	91 (50.84)
1 to 2 or equal to 2∗	47 (26.26)
Complicated SDNS/FRNS	29 (16.20)
Unable to determine	12 (6.70)
Time to first immunosuppressant (months)	16.40 ± 17.55
Steroid dose at relapse (mg/kg/d)	
Less than or equal to 0.3	60 (33.52)
0.3–0.75 or equal to 0.75	65 (36.31)
More than 0.75	38 (21.23)
Unable to determine	16 (8.94)
eGFR (mL/min·1.73 m^2^) in follow-up	
Decreased less than or equal to 20%	116 (64.80)
Decreased greater than 20%	49 (27.37)
Unable to determine	14 (7.82)

Notes: complicated SDNS/FRNS (steroid-dependent/frequent relapse nephrotic syndrome) patients receiving immunosuppressive agents still showed frequent relapses or steroid dependence after treatment; eGFR (estimated glomerular filtration rate) decrease in follow-up decreased average eGFR level in follow-up. “∗” refers to the course of taking additional immunosuppressants.

#### Steroid dose at relapse

2:171713702 (*GAD1*) was significantly associated with a greater steroid dose from relapse (>0.75) to first remission in patients with SDNS/FRNS (*OR* = 3.14; 95% CI: 0.97–9.87; *P* = 0.034; Table S16).

#### Total relapse times

Two SNPs, rs117014418 (*APOL4*) and rs139880713 (*FRG1JP*), were significantly associated with a higher relapse time in SDNS/FRNS (rs117014418, *n* > 3, OR = 0.40, 95% CI: 0.16–1.01, *P* = 0.043; rs139880713, *n* > 2, OR = 3.59, 95% CI: 1.07–15.62, *P* = 0.028; rs139880713, *n* > 3, OR = 0.29, 95% CI: 0.09–0.92, *P* = 0.025). In contrast, SDNS/FRNS patients carrying 2:171713702 in *GAD1* had lower total relapse times (*n* = 2, OR = 4.98; 95% CI: 1.12–46.13; *P* = 0.022; Table S16). These three SNPs were not in linkage disequilibrium with other major SNPs, indicating that these signals are responsible for driving the association. These results are consistent with the finding that 2:171713702 was significantly associated with a greater steroid dose at relapse. We hypothesize that SDNS/FRNS patients with 2:171713702 are more likely to receive attention from physicians and undergo more active management, such as immunosuppressant treatment and infection prevention.

#### eGFR at follow-up

**Figure 4:**
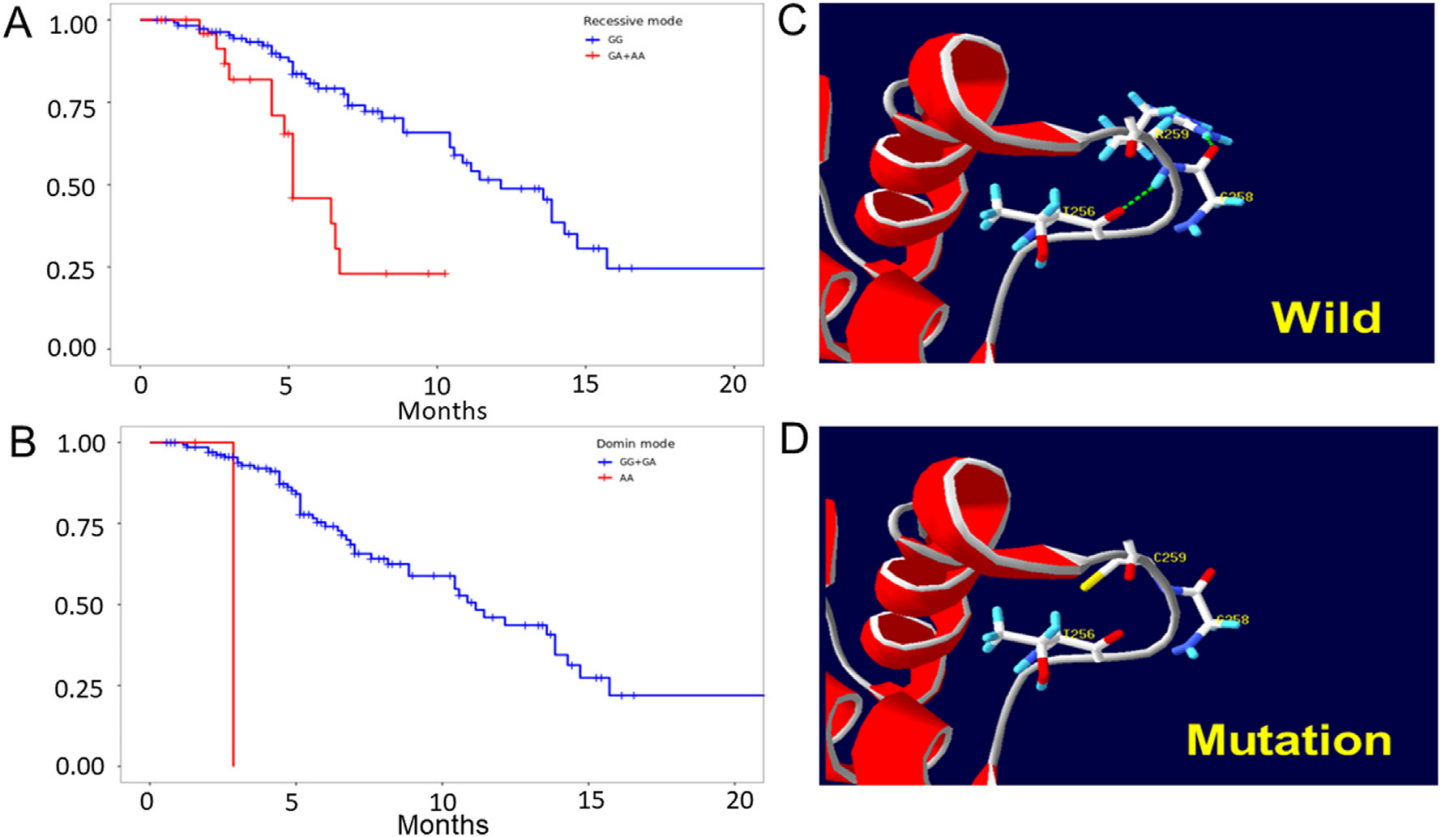
The structure and function changes of rs117014418 in *APOL4*. **(A)** Kaplan–Meier survival analysis concerning *APOL4* rs117014418 (upper panel, recessive; lower panel, dominant model). **(B)** rs117014418 *in silico* prediction model of APOL4 (upper panel, wild type; lower panel, mutation type).

Due to the complexity and poor prognosis of SDNS/FRNS, we sought to evaluate the association between significant SNPs and eGFR in follow-up. During the follow-up, no significant eGFR decline was observed in either the SSNSWR or SDNS/FRNS groups. Interestingly, rs117014418 in *APOL4* was significantly associated with a progressively decreasing eGFR greater than 20% compared with baseline in SDNS/FRNS patients according to a recessive model (*P* = 0.0001) and a dominant model (*P* = 0.00038) (Fig. 4A).

#### Protein structural prediction of variants

One SNP (rs117014418) in *APOL4* is a missense SNP. The APOL4 prediction model showed that in the wild-type protein, the 259th amino acid is arginine, which interacts with the 256th threonine and 258th glycine to form hydrogen bonds and maintain the three-dimensional structure. Conversely, in the mutant (rs117014418) protein, the 259th amino acid is cystine, which has no hydrogen bond interaction with the 256th threonine and 258th glycine, resulting in perturbation of the secondary structure of APOL4 (Fig. 4B). One SNP (rs774409792) is predicted to induce a synonymous mutation. The mutation 2:97909692 is predicted to be nonsynonymous with no hydrogen bond change. The remaining variants could not be predicted by electronic software, as shown in Table 4.

**Table 4:** Annotation and structural modeling of variant prediction.

SNP	Nucleotide	Amino acid	Region	Gene	Change
rs746236012	–	–	Intronic	*SLC9C2*	Unknown
2:97909692	c.4417G>A	p.V1473 M	Exonic	*ANKRD36*	Nonsynonymous
rs117962550	–	–	Intronic	*ALPG*	Unknown
2:171713702	–	–	Intronic	*GAD1*	Unknown
rs1047989	c.22C>A	p.L8 M	Exonic	*HLA-DQA1*	Nonsynonymous
rs139880713	c.1056-2A>G	–	ncRNA splicing	*FRG1JP*	Unknown
rs774409792	c.40923C>T	p.F13641F	Exonic	*MUC16*	Synonymous
rs117014418	c.766C>T	p.A259C	Exonic	*APOL4*	Missense

Notes: SNP, single-nucleotide polymorphism.

#### Protein–protein interaction network construction

We performed network analysis to investigate the connection between candidate genes, as shown in Table 2. The result was significant in the GWAS strategy comparison of SSNSWR *vs*. control, SDNS/FRNS *vs*. control, and SSNSWR *vs*. SDNS/FRNS. A subnetwork of 50 genes among these candidate genes was mapped using the STRING protein–protein interaction intersection database,^26^ and a minimum span tree of the subnetwork was performed to obtain a core skeleton. Figure S6 shows the hub genes and major interactions in the network. Interestingly, HLA-DQA1 and HLA-DQB1 appear to function together via gasdermin A (GSDMA), which was the top gene in the comparison group of SSNSWR versus SDNS/FRNS.

## Discussion

### HLA alleles and SSNS

SSNS may be an unintended consequence of the immune system responding to an infection, and this fits with the clinical observation that manifestations of the disease are typically preceded by infection. The involvement of the HLA system in SSNS has been confirmed. Our study identified a cluster of SNPs (rs1047989, rs1063322, rs1770, rs28383345, rs28383346, and rs9273471) in the *HLA-DQ* region that are associated with SDNS/FRNS. After conditional analysis, only rs1047989 was independent in driving the association in SDNS/FRNS. None of these significant SNPs in the *HLA-DQ* region were previously reported. One reported SNP, rs1140343, was also detected in our study (5.24 × 10^−7^) and was not in linkage disequilibrium with the SNPs found to be significant in this study, with r^2^ = 0.694 for the *HLA-DQA1* SNP (rs1047989). According to these results, the risk *HLA* locus that we identified may be particularly specific to the Chinese Han ethnicity with SSNS, providing an opportunity for further stratification and personalized medicine for patients with idiopathic nephrotic syndrome. In contrast, no previously reported *HLA* region-related susceptibility locus on chromosome 6 was found in SSNSWR. In clinical practice, patients with SSNSWR are sensitive to steroid treatment, with no relapse and a good prognosis. Overall, the differential distribution of *HLA* regions in subphenotypes of SSNS might help to explain the different disease characteristics between SSNSWR and SDNS/FRNS. We further speculate that these findings indirectly confirm the important role of *HLA* alleles in the development of other refractory nephropathies, such as SDNS/FRNS. Based on the appropriate number of patients with SSNSWR and SDNS/FRNS and a GWAS strategy, our study updates evidence that the *HLA* locus is associated with SSNS and may be a potential trigger for SDNS/FRNS.

### APOL gene series

The apolipoprotein gene series, a hot research topic outside the *HLA* region associated with GWAS strategies, has been identified as a risk factor for chronic kidney disease progression. The extent of the impact of apolipoprotein L4 (*APOL4*) remains unclear. Gregory A et al^27^ suggested that except for the *APOL1* G1 and G2 alleles, *APOL1-APOL4-MYH9*-region variants do not significantly contribute to the risk of end-stage kidney disease, and Adeyemo et al^8^ reported that the *APOL1* G1 and G2 alleles are strongly associated with SRNS but not SSNS in African–American patients. Interestingly, in our study, rs117014418 in an *APOL4* exon was associated with progressively decreasing eGFR during follow-up in both SSNSWR and SDNS/FRNS patients, even leading to an average higher relapse frequency in the latter. Compared with known variants of *APOL1-APOL4* in the GTEx database, we hypothesize that rs117014418 is associated with increased APOL4 expression, which may play a role in lipid exchange and transport throughout the body in SDNS/FRSN.

Overall, our study first identified genetic susceptibility variants associated with two major subphenotypes of steroid-sensitive nephrotic syndrome: SSNSWR and SDNS/FRNS. These two subphenotypes have extreme clinical characteristics that deserve further study. These risk variants are associated with *HLA* alleles and risk genes outside *HLA* regions, which broadens our understanding of the disease process of SSNS and will aid the prediction of the therapy response pattern and tailoring of immunosuppression.

### GSDMA

Protein–protein interaction network construction suggested that HLA-DQA1 and HLA-DQB1 work together through GSDMA. GSDMA belongs to the gasdermin family. The emerging roles of the gasdermin family include the regulation of various physiological and pathological processes, such as cell differentiation, coagulation, inflammation, and tumorigenesis.^28^ Deng et al suggested that GSDMA has the ability to control systemic infection and that *Gsdma1* genetic deficiency blunts mouse immune responses to group A Streptococcus, resulting in uncontrolled bacterial dissemination and death.^29^ RNA sequencing and genome-wide genotyping of monocyte-derived macrophages from patients with system sclerosis have revealed that the *GSDMA* rs3894194 risk variant contributes to several inflammatory pathways and system sclerosis susceptibility via up-regulation of glycolysis, hypoxia, and mTOR signaling and down-regulation of the IFN-γ response pathway.^30^ In addition, the GSDMA protein is detected in T lymphocytes.^31^ We speculate that genetic *GSDMA* variations may confer vulnerability to infections associated with HLA-DQA1 and HLA-DQB1, participating in the activation of a unique T-cell downstream pathway and regulating the immune response, resulting in the difference in renal damage among patients with SSNS.

### Limitations

This study has several limitations that should be considered. First, although the updated Schwartz equation is simpler and more accessible than the combined Schwartz equation in daily clinical practice, the updated Schwartz formula was adopted in this study to estimate eGFR, which may have led to a potential statistical error in the consideration of the severity of the illness in the disease course, especially for those with SDNS/FRNS. For example, the serum creatinine was measured during a relapse period which may potentially result in a lower eGFR. Second, there were limited patients with SSNSWR and SDNS/FRNS, and traditional GWAS replication was not conducted in our study. Instead, we added more complete clinical follow-up data for these SSNSWR and SDNS/FRNS patients. Therefore, this study is the first in which a GWAS strategy and bioinformatics analysis were performed to identify risk variant loci associated with subphenotype levels in SSNS and how these variants influence clinical phenotypes and treatment outcomes. In addition, we performed eQTL analysis of variants of interest with the GTEx expression dataset and protein structure of candidate genes. These analysis results enhanced the reliability of our conclusion. Because SSNS is a rare pediatric condition, our study design is reasonable and will contribute to clinical benefits in the future.^32^

## Conclusions

In summary, we describe several high-risk variants and genes associated with the two main subphenotypes of SSNS based on GWAS strategy analysis. The study confirms that the high-risk association between the *HLA* region and SSNS is potentially caused by frequent relapse or steroid dependence rather than other SSNS subphenotypes. These biomarkers will be immensely useful for risk stratification in children at the initial presentation of SSNS.

## Author contributions

H. Chan conducted the bioinformatics analysis and drafted the manuscript, worked together with H. Chan, L.W., F.N., J.D., B.Z., and J.C. from the branch centers of the National Clinical Research Center for Child Health and Disorders, and was involved in patient management. Q.L., X.R., and H.Y. proposed and conducted the study. L.W., F.N., J.D., X.Y., B.Z., S.F., J.C., X.G., H. Chi, H.L., X.C., X.L., J.J., and D.W. were responsible for data collection and data extraction. H.J., H.Y., G.Z., Y.C., and M.W. were responsible for the data analysis. L.W., F.N., J.D., X.Y., B.Z., S.F., J.C., X.G., H. Chi, H.L., X.C., and X.L. were responsible for enrolling the patients and collecting specimens.

## Conflict of interests

All authors declare no competing interests.

## Funding

This study was funded by the China National Natural Science Foundation (No. 81970618, 82170720, 82200788), China National Clinical Research Centre Foundation (No. NCRC-2019-GP-02), Science and Technology Research Project of Chongqing Education Commission of China (No. KJZD-M201900401), Chongqing Science and Health Joint Medical Research Project (China) (No. 2023GGXM001), and 10.13039/501100012166National Key R&D Program of China (No. 2022YFC2705101). The authors declare that they have no relevant financial interests.
