# Supplementary material for: A genomic association study revealing subphenotypes of childhood steroid-sensitive nephrotic syndrome in a larger genomic sequencing cohort
Source: Genes Dis. 2023 Sep 27;11(4):101126. doi: 10.1016/j.gendis.2023.101126 (PMC10978544; doi:10.1016/j.gendis.2023.101126)
Supplement: Multimedia component 1 [file mmc1.docx]

Supplementary Information

**A genomic association study revealing subphenotypes of childhood steroid-sensitive nephrotic syndrome in a larger genomic sequencing cohort**

Supplementary Figure 1: Principal component analysis of the patients.

Supplementary Figure 2: Plots of the principal components (PC1 and PC2) of the genotypes projected against 1000 Genomes population reference samples.

Supplementary Figure 3: Plot of LD decay of chromosomes.

Supplementary Figure 4: QQ plots of the discovery stage.

Supplementary Figure 5: Rank normalized, adjusted glomerular expression for rs1047989.

Supplementary Figure 6: PPI network construction for the hub genes.

Supplementary Table 1: Protocol of genomic DNA extraction

Supplementary Table 2: Flow table of selection process for SNPs after genotyping.

Supplementary Table 3: The sequencing depth of each sample.

Supplementary Table 4: Details of the main genotyped SNPs between the SSNSWR and SDNS/FRNS groups.

Supplementary Table 5:Genome-wide significant loci from the SAIGE tests.

Supplementary Table 6: Top genes in rare variant analysis.

Supplementary Table 7: Distribution of genotypes and genetic model analysis for rs746236012.

Supplementary Table 8: Distribution of genotypes and genetic model analysis for rs117962550.

Supplementary Table 9: Supplementary Table 9: Distribution of genotypes and genetic model analysis for 2:97909692.

Supplementary Table 10: Distribution of genotypes and genetic model analysis for 2:171713702.

Supplementary Table 11: Distribution of genotypes and genetic model analysis for rs1047989.

Supplementary Table 12: Distribution of genotypes and genetic model analysis for rs139880713.

Supplementary Table 13: Distribution of genotypes and genetic model analysis for rs774409792.

Supplementary Table 14: Distribution of genotypes and genetic model analysis for rs117014418.

Supplementary Table 15: Clinical criteria for inclusion.

Supplementary Table 16:Subgroup analysis of the potential SNPs and clinical characteristics.

Supplementary Methods: Genotyping and quality control for WES data.

Supplementary Figure 1: Principal component analysis of the patients: A. SSNSWR and SDNS/FRNS; B. INS patients and healthy controls.


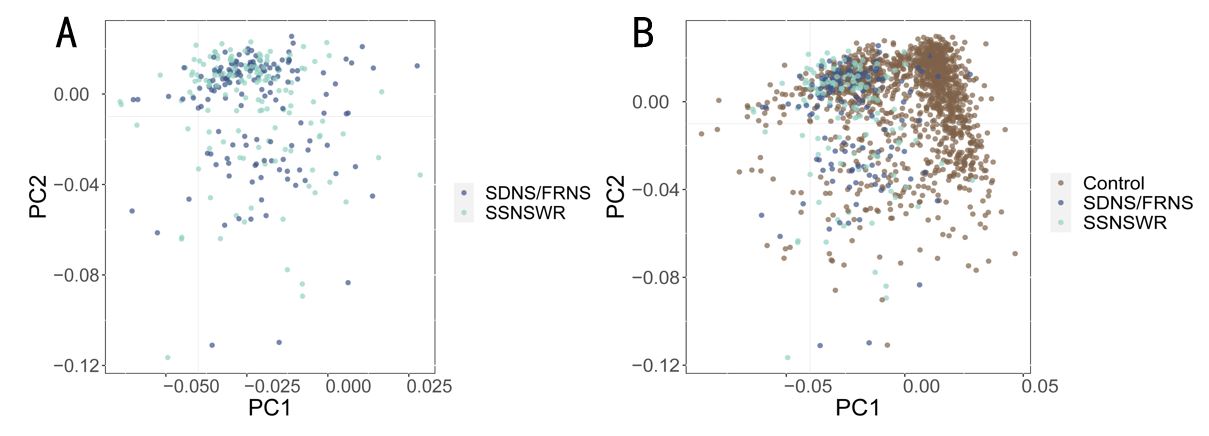


Supplementary Figure 2: Plots of principal components (PC1 and PC2) of the

genotypes projected against 1000 Genomes population reference samples. (AFR= Africa, ASN = Asia (East Asia); AMR =the Americas; EUR = Europe; CHB=Han Chinese in Beijing; CHS=Southern Han Chinese; JPT=Japanese in Tokyo; OWN=Patients).


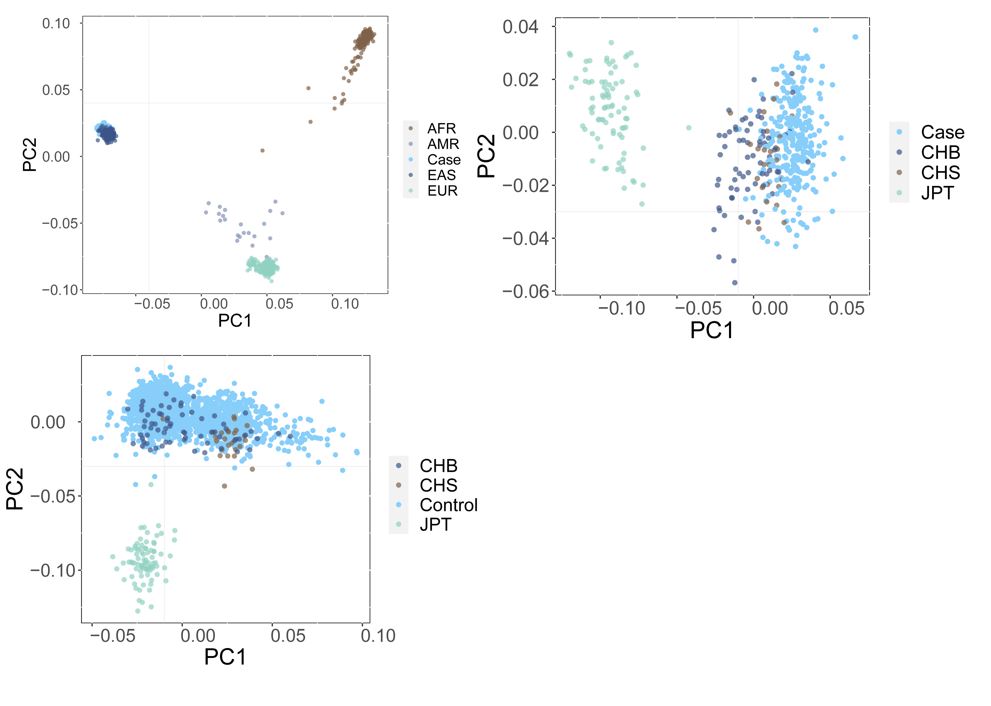


Supplementary Figure 3: Plot of LD decay for chromosomes.


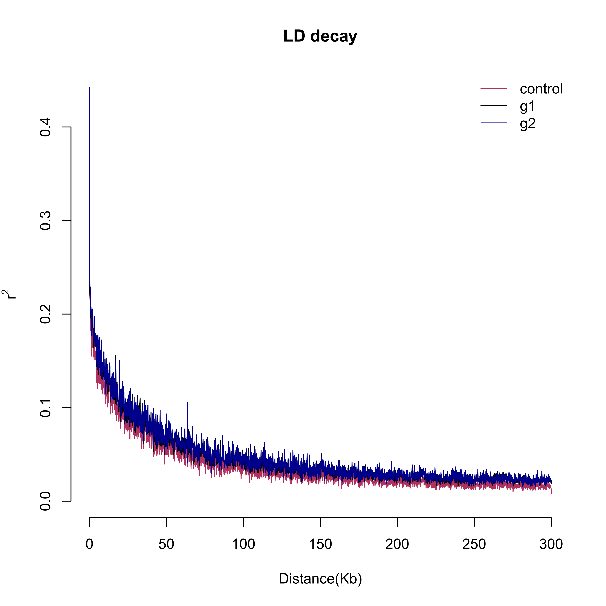

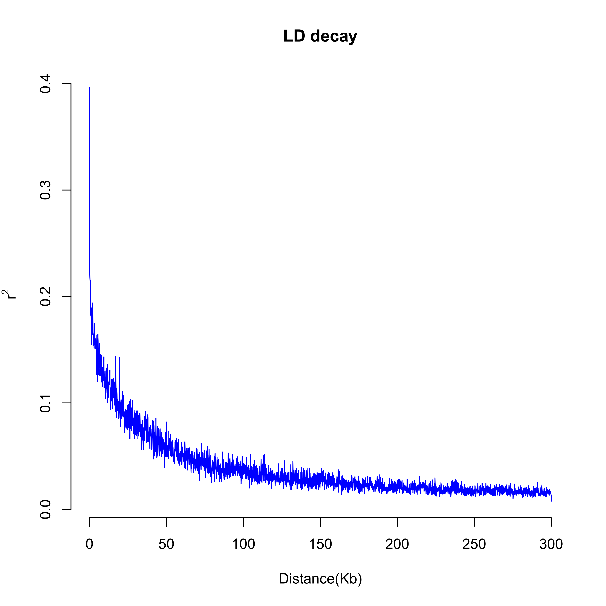


**SSNSWR**

**Merge**


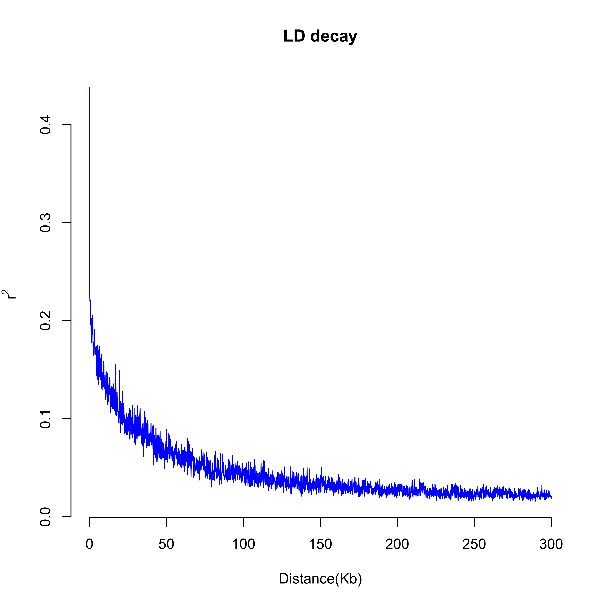

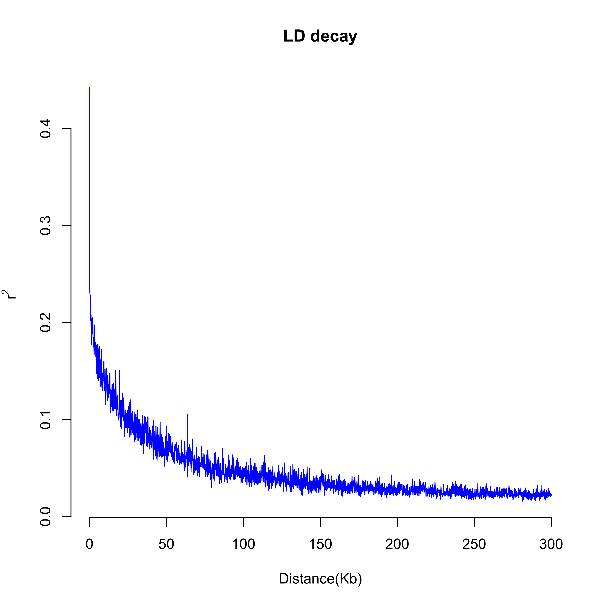


**SSNSWR+SDNS/FRNS**

**SDNS/FRNS**

Supplementary Figure 4: QQ plots for the discovery stage: (A) SSNSWR vs Control (g1), (B) SDNS/FRNS vs Control (g2), and (C) SSNSWR vs SDNS/FRNS (g1_g2).


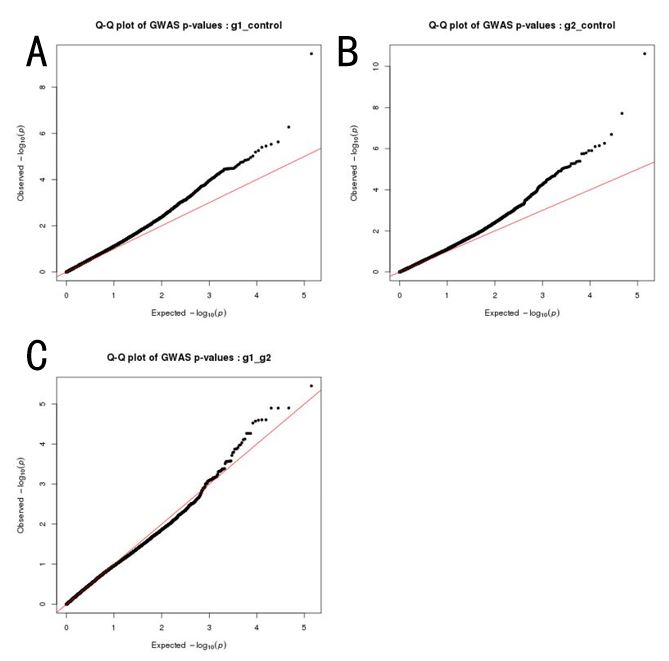


G1_Control: λ=1.1144，Intercept=1.0806

G2_Control: λ=1.1364，Intercept=1.0414

G1_G2_CK: λ=1.0988，Intercept=1.043

Supplementary Figure 5: Rank normalized, adjusted glomerular expression for rs1047989.


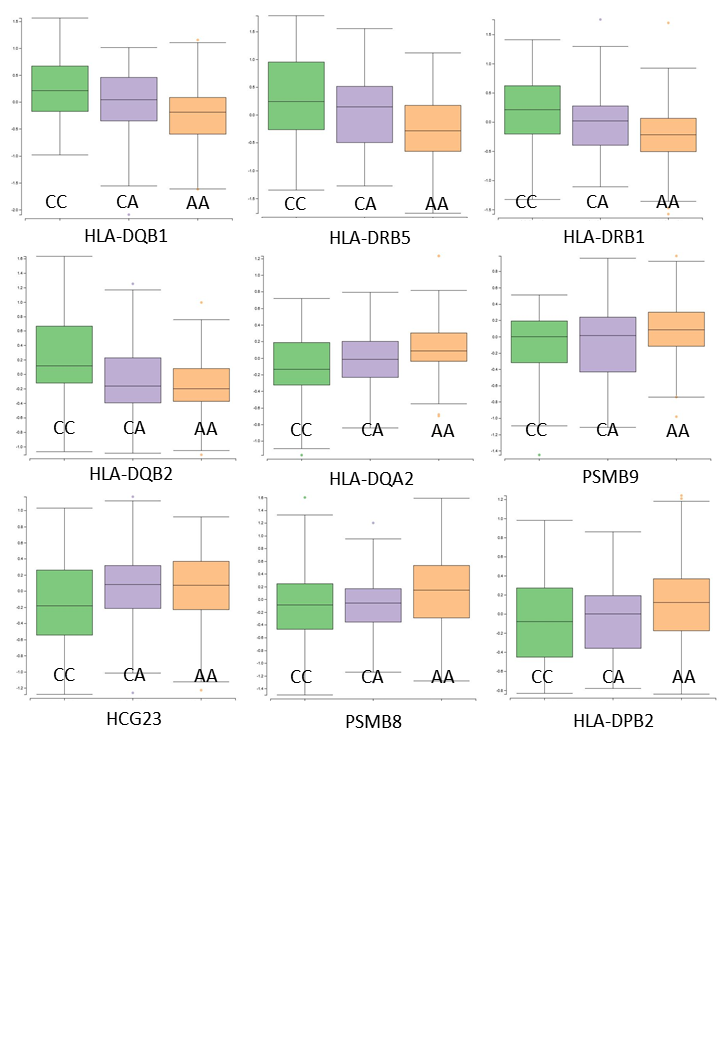


Supplementary Figure 6: PPI network construction for the hub genes.


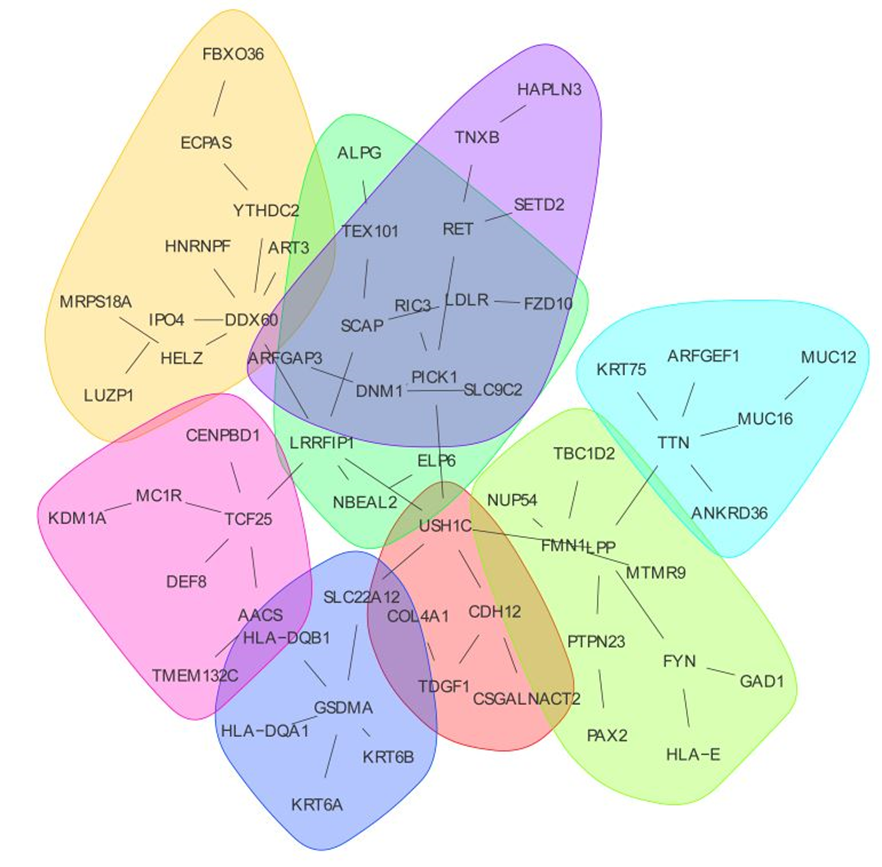


Supplementary Table 1: Protocol of genomic DNA extraction

| Step | Experimental operation |
| --- | --- |
| 1 | Pipet 20µl QIAGEN Protease stock solution into the bottom of the collection microtubes. |
| 2 | Add samples to the collection microtubes by touching the insides of the tubes without wetting the rims. Use either 200µl whole blood, plasma, serum, or body fluids per tube, or up to 5x106 lymphocytes or cultured cells in 200µl PBS per tube. Use the Plate Register provided to record the locations of the samples. |
| 3 | Add 200µl Buffer AL to each sample, taking care not to wet the rims of the collection microtubes. Seal the tubes using the caps for collection microtubes provided. |
| 4 | Cover the rack with the plastic cover supplied, and mix thoroughly by shaking vigorously for 15s. |
| 5 | Centrifuge briefly at 3000 rpm to collect any solution from the caps. |
| 6 | Incubate at 70°C for at least 10 min in an incubator or oven. |
| 7 | Centrifuge briefly at 3000 rpm to collect any lysate from the caps. |
| 8 | Remove the caps and add 200µl ethanol (96-100%) to each tube. |
| 9 | Seal the tubes using new caps for collection microtubes. Shake vigorously for 15s. |
| 10 | Centrifuge briefly at 3000 rpm to collect any solution from the caps. |
| 11 | Place QIAamp 96 plates on top of an S-Block. Mark the plate for later identification. |
| 12 | Carefully apply the mixture from step 8 (620µl per collection microtube) to the QIAamp 96 plate. |
| 13 | Seal the QIAamp 96 plate with an AirPore Tape sheet. Load the S-Block and QIAamp 96 plate onto the carrier, then place it in the rotor bucket. Centrifuge at 6000 rpm for 4 min. |
| 14 | Remove the tape. Carefully add 500µl Buffer AW1 to each well. |
| 15 | Seal the QIAamp 96 plate with a new AirPore Tape sheet. Centrifuge at 6000 rpm for 2 min. |
| 16 | Remove the tape. Carefully add 500µl Buffer AW2 to each well. |
| 17 | Centrifuge at 6000 rpm for 15 min. |
| 18 | Place the QIAamp 96 plate on top of a rack of elution microtubes (provided). |
| 19 | To elute DNA, add 200µl Buffer AE or distilled water, equilibrated to room temperature, to each well using a multichannel pipet. Seal the QIAamp 96 plate with a new AirPore tape sheet and incubate for 1 min at room temperature. Centrifuge at 6000 rpm for 4 min. Seal the wells of the microtubes for storage using the caps for elution microtubes provided. |

Supplementary Table 2: Flow table of selection process for SNPs after genotyping.

| Total number of SNPs after genotyping (n) | SSNSWR  60251965 (SNPs) | SDNS/FRNS  60249203 (SNPs) | SSNSWR and SDNS/FRNS  60268469 (SNPs) | Control  60310932 (SNPs) |
| --- | --- | --- | --- | --- |
| Step 1: excluded call rate<98% | 45629276 | 56980343 | 45814137 | 56813777 |
| Step 2: excluded sex chromosome and mitochondria | 44146253 | 55244420 | 44331212 | 55015622 |
| Step 3: excluded MAF<1% | 89007 | 121714 | 86189 | 98342 |
| Step 4: Hardy-Weinberg equilibrium | NA | NA | NA | 55008097 |
| Remaining | 89007 | 121714 | 86189 | 98342 |
| Merge | 76423 | | 63465 | |

Supplementary Table 3: The sequencing depth of each sample.

| P1 | 213.42 | | P26 | | 112.44 | | P51 | | 123.6 | | P76 | | 130.52 | | P101 | | 140.72 | | P126 | | 147.31 | | P151 | 152.96 | | P176 | 162.23 |
| --- | --- | --- | --- | --- | --- | --- | --- | --- | --- | --- | --- | --- | --- | --- | --- | --- | --- | --- | --- | --- | --- | --- | --- | --- | --- | --- | --- |
| P2 | 87.51 | | P27 | | 112.66 | | P52 | | 123.9 | | P77 | | 130.97 | | P102 | | 140.93 | | P127 | | 148.58 | | P152 | 153.34 | | P177 | 162.4 |
| P3 | 90.19 | | P28 | | 112.88 | | P53 | | 124.13 | | P78 | | 131.31 | | P103 | | 141.2 | | P128 | | 148.74 | | P153 | 153.42 | | P178 | 162.45 |
| P4 | 92.61 | | P29 | | 113.37 | | P54 | | 124.72 | | P79 | | 131.4 | | P104 | | 142.03 | | P129 | | 149.07 | | P154 | 153.87 | | P179 | 162.67 |
| P5 | 100.47 | | P30 | | 113.44 | | P55 | | 124.76 | | P80 | | 131.49 | | P105 | | 142.11 | | P130 | | 149.27 | | P155 | 153.99 | | P180 | 162.75 |
| P6 | 100.58 | | P31 | | 113.69 | | P56 | | 124.87 | | P81 | | 131.82 | | P106 | | 142.15 | | P131 | | 149.33 | | P156 | 154.49 | | P181 | 163.05 |
| P7 | 102.12 | | P32 | | 113.7 | | P57 | | 125.14 | | P82 | | 131.85 | | P107 | | 142.2 | | P132 | | 149.59 | | P157 | 154.84 | | P182 | 163.63 |
| P8 | 103.05 | | P33 | | 115 | | P58 | | 125.64 | | P83 | | 132.24 | | P108 | | 142.24 | | P133 | | 149.81 | | P158 | 155.53 | | P183 | 163.66 |
| P9 | 104.76 | | P34 | | 116.22 | | P59 | | 125.88 | | P84 | | 132.38 | | P109 | | 142.53 | | P134 | | 149.82 | | P159 | 155.95 | | P184 | 163.77 |
| P10 | 105.79 | | P35 | | 116.43 | | P60 | | 126.32 | | P85 | | 132.86 | | P110 | | 142.55 | | P135 | | 149.82 | | P160 | 155.95 | | P185 | 163.99 |
| P11 | 105.95 | | P36 | | 116.63 | | P61 | | 127.29 | | P86 | | 132.89 | | P111 | | 142.62 | | P136 | | 150.14 | | P161 | 156 | | P186 | 164.01 |
| P12 | 106.17 | | P37 | | 116.94 | | P62 | | 127.42 | | P87 | | 133.62 | | P112 | | 142.69 | | P137 | | 150.18 | | P162 | 156.81 | | P187 | 164.19 |
| P13 | 106.36 | | P38 | | 118.8 | | P63 | | 127.51 | | P88 | | 133.9 | | P113 | | 143.33 | | P138 | | 150.41 | | P163 | 156.92 | | P188 | 164.38 |
| P14 | 106.66 | | P39 | | 118.86 | | P64 | | 127.88 | | P89 | | 134.05 | | P114 | | 143.5 | | P139 | | 150.88 | | P164 | 157.45 | | P189 | 164.51 |
| P15 | 106.68 | | P40 | | 119.2 | | P65 | | 128.14 | | P90 | | 134.1 | | P115 | | 143.66 | | P140 | | 151.13 | | P165 | 158.15 | | P190 | 164.67 |
| P16 | 107.97 | | P41 | | 119.47 | | P66 | | 128.29 | | P91 | | 134.28 | | P116 | | 143.95 | | P141 | | 151.15 | | P166 | 158.64 | | P191 | 164.92 |
| P17 | 108.28 | | P42 | | 120.21 | | P67 | | 128.53 | | P92 | | 134.66 | | P117 | | 144.09 | | P142 | | 151.33 | | P167 | 159.19 | | P192 | 165.15 |
| P18 | 108.66 | | P43 | | 120.77 | | P68 | | 128.63 | | P93 | | 134.91 | | P118 | | 144.31 | | P143 | | 151.45 | | P168 | 159.2 | | P193 | 165.2 |
| P19 | 109.03 | | P44 | | 121.66 | | P69 | | 128.97 | | P94 | | 135.29 | | P119 | | 144.43 | | P144 | | 151.47 | | P169 | 159.3 | | P194 | 165.34 |
| P20 | 109.55 | | P45 | | 121.7 | | P70 | | 129.04 | | P95 | | 135.3 | | P120 | | 145.23 | | P145 | | 151.5 | | P170 | 159.73 | | P195 | 165.49 |
| P21 | 111.34 | | P46 | | 121.74 | | P71 | | 129.81 | | P96 | | 137.83 | | P121 | | 145.29 | | P146 | | 151.66 | | P171 | 159.79 | | P196 | 165.81 |
| P22 | 111.59 | | P47 | | 121.8 | | P72 | | 129.94 | | P97 | | 138.06 | | P122 | | 145.38 | | P147 | | 152.01 | | P172 | 160.46 | | P197 | 166.03 |
| P23 | 112.22 | | P48 | | 122.1 | | P73 | | 129.94 | | P98 | | 138.13 | | P123 | | 145.69 | | P148 | | 152.36 | | P173 | 160.65 | | P198 | 166.2 |
| P24 | 112.31 | | P49 | | 122.38 | | P74 | | 130.2 | | P99 | | 138.52 | | P124 | | 146.03 | | P149 | | 152.51 | | P174 | 161.16 | | P199 | 166.24 |
| P25 | 112.32 | | P50 | | 122.51 | | P75 | | 130.38 | | P100 | | 139.92 | | P125 | | 146.81 | | P150 | | 152.69 | | P175 | 161.75 | | P200 | 166.54 |
| P201 | | 166.55 | | P226 | | 172.24 | | P251 | | 180.39 | | P276 | | 192.15 | | P301 | | 204.63 | | P326 | | 237.39 | | |  |  |  |
| P202 | | 166.67 | | P227 | | 172.61 | | P252 | | 180.87 | | P277 | | 192.33 | | P302 | | 204.95 | | P327 | | 242.15 | | |  |  |  |
| P203 | | 167.01 | | P228 | | 172.67 | | P253 | | 181.04 | | P278 | | 192.65 | | P303 | | 205.82 | | P328 | | 245.82 | | |  |  |  |
| P204 | | 167.11 | | P229 | | 172.7 | | P254 | | 181.23 | | P279 | | 193.17 | | P304 | | 205.95 | | P329 | | 246.29 | | |  |  |  |
| P205 | | 167.48 | | P230 | | 172.82 | | P255 | | 182.23 | | P280 | | 193.7 | | P305 | | 206.35 | | P330 | | 247.29 | | |  |  |  |
| P206 | | 167.58 | | P231 | | 173.71 | | P256 | | 183.12 | | P281 | | 194.21 | | P306 | | 206.66 | | P331 | | 261.29 | | |  |  |  |
| P207 | | 167.59 | | P232 | | 174.03 | | P257 | | 183.26 | | P282 | | 194.23 | | P307 | | 207.97 | | P332 | | 262.26 | | |  |  |  |
| P208 | | 167.79 | | P233 | | 174.06 | | P258 | | 184.25 | | P283 | | 194.96 | | P308 | | 208.18 | | P333 | | 280.37 | | |  |  |  |
| P209 | | 167.97 | | P234 | | 174.38 | | P259 | | 184.91 | | P284 | | 195.36 | | P309 | | 208.83 | | P334 | | 287.08 | | |  |  |  |
| P210 | | 168.18 | | P235 | | 174.48 | | P260 | | 185.64 | | P285 | | 195.45 | | P310 | | 209.36 | | P335 | | 313.8 | | |  |  |  |
| P211 | | 168.34 | | P236 | | 174.61 | | P261 | | 185.64 | | P286 | | 195.5 | | P311 | | 209.37 | | P336 | | 107.03 | | |  |  |  |
| P212 | | 168.58 | | P237 | | 174.85 | | P262 | | 185.8 | | P287 | | 195.53 | | P312 | | 209.92 | | P337 | | 111.56 | | |  |  |  |
| P213 | | 168.7 | | P238 | | 175.31 | | P263 | | 186.39 | | P288 | | 195.82 | | P313 | | 210.11 | | P338 | | 116.96 | | |  |  |  |
| P214 | | 169.07 | | P239 | | 175.35 | | P264 | | 186.55 | | P289 | | 196.36 | | P314 | | 211.26 | | P339 | | 122.7 | | |  |  |  |
| P215 | | 169.17 | | P240 | | 175.77 | | P265 | | 186.69 | | P290 | | 196.71 | | P315 | | 211.3 | | P340 | | 123.12 | | |  |  |  |
| P216 | | 169.25 | | P241 | | 176.05 | | P266 | | 186.73 | | P291 | | 197.75 | | P316 | | 212.69 | | P341 | | 138.47 | | |  |  |  |
| P217 | | 169.26 | | P242 | | 177.02 | | P267 | | 186.91 | | P292 | | 197.92 | | P317 | | 214.93 | | P342 | | 156.97 | | |  |  |  |
| P218 | | 170.21 | | P243 | | 177.54 | | P268 | | 186.95 | | P293 | | 198.35 | | P318 | | 217.02 | | P343 | | 158.64 | | |  |  |  |
| P219 | | 170.31 | | P244 | | 177.84 | | P269 | | 187.81 | | P294 | | 198.65 | | P319 | | 219.82 | | P344 | | 166.8 | | |  |  |  |
| P220 | | 170.59 | | P245 | | 177.94 | | P270 | | 187.91 | | P295 | | 201.46 | | P320 | | 224.53 | |  | |  | | |  |  |  |
| P221 | | 170.77 | | P246 | | 178.78 | | P271 | | 188.62 | | P296 | | 201.54 | | P321 | | 225.48 | |  | |  | | |  |  |  |
| P222 | | 170.86 | | P247 | | 179.36 | | P272 | | 189.56 | | P297 | | 202.15 | | P322 | | 227.89 | |  | |  | | |  |  |  |
| P223 | | 171.12 | | P248 | | 179.71 | | P273 | | 191.24 | | P298 | | 202.42 | | P323 | | 229.15 | |  | |  | | |  |  |  |
| P224 | | 171.28 | | P249 | | 180.16 | | P274 | | 191.26 | | P299 | | 203.37 | | P324 | | 233.63 | |  | |  | | |  |  |  |
| P225 | | 171.96 | | P250 | | 180.23 | | P275 | | 191.36 | | P300 | | 203.8 | | P325 | | 235.2 | |  | |  | | |  |  |  |

Supplementary Table 4: Details of the main genotyped SNPs between SSNSWR and SDNS/FRNS.

| **CHR** | **SNP** | **BP** | **A1** | **OR** | **L95** | **U95** | **P_Value** | **SSNSWR** | **SDNS/FRNS** | **Func.refGene** | **Gene.refGene** |
| --- | --- | --- | --- | --- | --- | --- | --- | --- | --- | --- | --- |
| chr17 | rs7212938 | 38122680 | T | 0.5 | 0.3617 | 0.6913 | 2.73E-05 | 120/174 | 137/168 | exonic | **GSDMA** |
| chr17 | rs9914973 | 38122708 | C | 0.4997 | 0.3593 | 0.695 | 3.75E-05 | 94/174 | 125/170 | intronic | **GSDMA** |
| chr2 | rs3739038 | 238672703 | G | 1.881 | 1.364 | 2.592 | 0.000116 | 144/174 | 106/170 | exonic | LRRFIP1 |
| chr16 | rs12597913 | 90021685 | G | 2.001 | 1.406 | 2.849 | 0.000118 | 101/174 | 62/170 | intronic | DEF8 |
| chr2 | rs3213868 | 238668802 | G | 1.864 | 1.351 | 2.571 | 0.000149 | 144/174 | 106/170 | exonic | LRRFIP1 |
| chr16 | rs4785755 | 90037828 | A | 1.944 | 1.377 | 2.743 | 0.000158 | 107/174 | 70/170 | exonic | CENPBD1 |
| chr16 | rs8166 | 90025640 | C | 1.971 | 1.386 | 2.803 | 0.00016 | 99/174 | 61/170 | exonic | DEF8 |
| chr12 | rs10848026 | 130647709 | C | 1.82 | 1.33 | 2.491 | 0.000184 | 143/174 | 109/170 | exonic | FZD10 |
| chr2 | rs499449 | 230841074 | T | 0.5401 | 0.39 | 0.7479 | 0.000209 | 106/174 | 127/170 | splicing | FBXO36 |
| chr16 | rs2293586 | 89962394 | G | 1.936 | 1.362 | 2.752 | 0.00023 | 101/174 | 66/170 | splicing | TCF25 |

Supplementary Table 5:Genome-wide significant loci from the SAIGE tests.

| **CHR** | **SNP** | **BP** | **Allele1** | **Allele2** | **BETA** | **SE** | **Tstat** | **var** | **p.value** |
| --- | --- | --- | --- | --- | --- | --- | --- | --- | --- |
| **SSNSWR vs. Control** | |  |  |  |  |  |  |  |  |
| 2 | rs117962550 | 233271731 | C | A | 1.83282 | 0.334187 | 18.9906 | 10.3614 | 4.15E-08 |
| 11 | rs67341709 | 1018496 | G | T | 2.03031 | 0.395156 | 14.919 | 7.34814 | 2.78E-07 |
| 2 | 2:97909692 | 97909692 | G | A | 1.08987 | 0.216314 | 24.8265 | 22.7794 | 4.70E-07 |
| **SDNS/FRNS vs. Control** | |  |  |  |  |  |  |  |  |
| 6 | rs1047989 | 32605257 | A | C | -0.8907 | 0.129055 | -54.3271 | 60.994 | 5.14E-12 |
| 1 | 1:156640671 | 156640671 | C | C | 1.63932 | 0.31688 | 18.1577 | 11.0764 | 2.30E-07 |
| 6 | rs1049123 | 32627794 | C | T | 1.86296 | 0.36493 | 17.0554 | 9.15499 | 3.31E-07 |
| 6 | rs1770 | 32627833 | A | G | 0.680081 | 0.133852 | 38.3007 | 56.3179 | 3.76E-07 |
| 6 | rs1063322 | 32629935 | G | C | -0.66335 | 0.132759 | -38.1067 | 57.4458 | 5.83E-07 |
| **SSNSWR vs. SDNS/FRNS** | |  |  |  |  |  |  |  |  |
| 17 | rs7212938 | 38122680 | G | T | -0.67165 | 0.157894 | -26.5267 | 39.4947 | 2.10E-05 |
| 17 | rs9914973 | 38122708 | T | C | -0.66994 | 0.160898 | -25.5003 | 38.0637 | 3.13E-05 |
| 10 | rs4405241 | 1.03E+08 | A | C | 1.43822 | 0.358516 | 9.98373 | 6.94172 | 6.03E-05 |

Supplementary Table 6: Top genes in rare variant analysis.

| **CHR** | **BEGIN** | **END** | **GENE ID** | **NUM_ALL_VARS** | **NUM_PASS_VARS** | **NUM_SING_VARS** | **P.value** |
| --- | --- | --- | --- | --- | --- | --- | --- |
| **SSNSWR vs. Control** | |  |  |  |  |  |  |
| 3 | 131624173 | 131624178 | CPNE4 | 2 | 2 | 2 | **1.28E-30** |
| 3 | 47882496 | 47887793 | DHX30 | 2 | 2 | 2 | 2.37E-10 |
| 11 | 6519515 | 6592456 | DNHD1 | 37 | 35 | 8 | 2.84E-10 |
| 3 | 160138655 | 160148452 | SMC4 | 3 | 3 | 0 | 4.30E-09 |
| 2 | 74900905 | 74907179 | SEMA4F | 5 | 5 | 2 | 1.26E-08 |
| 1 | 31212687 | 31212687 | LAPTM5 | 1 | 1 | 0 | 2.58E-08 |
| 2 | 179393398 | 179667000 | TTN | 200 | 195 | 32 | 3.27E-08 |
| **SDNS/FRNS vs. Control** | |  |  |  |  |  |  |
| 3 | 131624173 | 131624178 | CPNE4 | 2 | 2 | 0 | **1.32E-10** |
| 6 | 72596754 | 73017051 | RIMS1 | 7 | 6 | 1 | 1.10E-08 |
| 6 | 52288769 | 52357072 | EFHC1 | 9 | 9 | 0 | 2.40E-08 |
| 11 | 14535162 | 14535162 | PSMA1 | 1 | 1 | 0 | 2.40E-08 |
| 11 | 14535162 | 14535162 | RP11-140L24.4 | 1 | 1 | 0 | 2.40E-08 |
| 8 | 79578393 | 79578393 | ZC2HC1A | 1 | 1 | 0 | 2.46E-08 |
| 15 | 59429637 | 59564608 | MYO1E | 4 | 4 | 1 | 2.67E-08 |
| 11 | 114272556 | 114278386 | RBM7 | 2 | 2 | 0 | 4.86E-08 |
| 11 | 114272556 | 114272556 | RP11-212D19.4 | 1 | 1 | 0 | 6.06E-08 |
| 1 | 167064097 | 167097496 | DUSP27 | 11 | 10 | 1 | 6.29E-08 |
| 17 | 9281904 | 9471780 | STX8 | 4 | 3 | 0 | 6.78E-08 |

Supplementary Table 7: Distribution of genotypes and genetic model analysis for rs746236012.

|  | Genotype | Case (%) | Control (%) | *P* value | OR (95%CI) |
| --- | --- | --- | --- | --- | --- |
| Allele | A | 24(6.90) | 75(2.09) | 4.81E-08 | 3.464(2.157,5.562) |
|  | G | 324(93.10) | 3507(97.91) |  |  |
| Genotype | AA | 0(0.00) | 0(0.00) | - | - |
|  | AG | 24(13.79) | 75(4.19) | 8.85E-08 | 3.661(2.245,5.970) |
|  | GG | 150(86.21) | 1716(95.81) | - | - |
| Recessive model | AA vs. (AG+GG) | 0(0.00)/174(100.00) | 0(0.00)/1791(100.00) | - | - |
| Dominant model | (AA+AG) vs. GG | 24(13.79)/150(86.21) | 75(4.19)/1716(86.21) | 8.85E-08 | 3.661(2.245,5.970 |
| Additive model | AA vs. AG vs. GG | 0(0.00)/24(13.79)/150(86.21) | 0(0.00)/75(4.19)/1716(95.81) | 1.02E-07 | 4.395(2.548,7.58) |

Supplementary Table 8: Distribution of genotypes and genetic model analysis for rs117962550.

|  | Genotype | Case (%) | Control (%) | *P* value | OR (95%CI) |
| --- | --- | --- | --- | --- | --- |
| Allele | A | 36(10.34) | 126(3.50) | 8.26E-10 | 3.178(2.156,4.684) |
|  | C | 312(89.66) | 3470(96.50) |  |  |
| Genotype | AA | 0(0.00) | 0(0.00) | - | - |
|  | AC | 36(20.69) | 126(7.01) | 8.72E-10 | 3.462(2.300,5.211) |
|  | CC | 138(79.31) | 1672(92.99) | - |  |
| Recessive model | AA vs. (AC+CC) | 0(0.00)/174(100.00) | 0(0.00)/1798(100.00) | - |  |
| Dominant model | (AA+AC) vs CC | 36(20.69)/138(79.31) | 126(7.01)/1672(79.31) | 8.72E-10 | 3.462(2.300,5.211) |
| Additive model | AA vs AC vs CC | 0(0.00)/36(20.69)/138(79.31) | 0(0.00)/126(7.01)/1672(92.99) | 7.17E-10 | 4.134(2.632,6.492) |

Supplementary Table 9: Distribution of genotypes and genetic model analysis for 2:97909692.

|  | Genotype | Case (%) | Control (%) | *P* value | OR (95%CI) |
| --- | --- | --- | --- | --- | --- |
| Allele | A | 68(19.54) | 324(9.04) | 4.20E-10 | 2.445(1.832,3.263) |
|  | G | 280(80.46) | 3262(90.96) |  |  |
| Genotype | AA | 1(0.57) | 7(0.39) | - | - |
|  | AG | 66(37.93) | 310(17.29) | 1 | 1.971(0.240,16.165) |
|  | GG | 107(61.49) | 1476(82.32) | 6.59E-11 | 2.937(2.111,4.086) |
| Recessive model | AA vs (AG+GG) | 1(0.57)/173(99.43) | 7(0.39)/1786(99.43) | 1 | 1.475(0.180,12.057) |
| Dominant model | (AA+AG) vs GG | 67(38.51)/107(61.49) | 317(17.68)/1476(61.49) | 7.18E-11 | 2.916(2.099,4.050) |
| Additive model | AA vs AG vs GG | 1(0.57)/66(37.93)/107(61.49) | 7(0.39)/310(17.29)/1476(82.32) | 3.37E-08 | 2.617(1.86,3.682) |

Supplementary Table 10: Distribution of genotypes and genetic model analysis for 2:171713702.

|  | Genotype | Case (%) | Control (%) | *P* value | OR (95%CI) |
| --- | --- | --- | --- | --- | --- |
| Allele | T | 21(6.18) | 46(1.30) | 4.05E-11 | 5.009(2.952,8.499) |
|  | C | 319(93.82) | 3500(98.70) |  |  |
| Genotype | TT | 0(0.00) | 0(0.00) | - | - |
|  | TC | 21(12.35) | 46(2.59) | 1.19E-10 | 5.291(3.075,9.104) |
|  | CC | 149(87.65) | 1727(97.41) | - | 1 |
| Recessive model | TT vs (TC+CC) | 0(0.00)/170(100.00) | 0(0.00)/1773(100.00) | - | - |
| Dominant model | (TT+TC) vs CC | 21(12.35)/149(87.65) | 46(2.59)/1727(87.65) | 1.19E-10 | 5.291(3.075,9.104) |
| Additive model | TT vs TC vs CC | 0(0.00)/21(12.35)/149(87.65) | 0(0.00)/46(2.59)/1727(97.41) | 1.37E-08 | 5.704(3.127,10.4) |

Supplementary Table 11: Distribution of genotypes and genetic model analysis for rs1047989.

|  | Genotype | Case (%) | Control (%) | *P* value | OR (95%CI) |
| --- | --- | --- | --- | --- | --- |
| Allele | C | 107(31.47) | 1693(47.08) | 3.34E-08 | 0.516(0.407,0.655) |
|  | A | 233(68.53) | 1903(52.92) |  |  |
| Genotype | CC | 20(11.76) | 394(21.91) | 2.46E-06 | 0.305(0.184,0.506) |
|  | CA | 67(39.41) | 905(50.33) | 2.99E-06 | 0.445(0.317,0.625) |
|  | AA | 83(48.82) | 499(27.75) | - | 1 |
| Recessive model | CC vs (CA+AA) | 20(11.76)/150(88.24) | 394(21.91)/1404(88.24) | 0.002658 | 0.475(0.294,0.768) |
| Dominant model | (CC+CA) vs AA | 87(51.18)/83(48.82) | 1299(72.25)/499(48.82) | 1.46E-08 | 0.403(0.293,0.553) |
| Additive model | CC vs CA vs AA | 20(11.76)/67(39.41)/83(48.82) | 394(21.91)/905(50.33)/499(27.75) | 1.25E-05 | 0.5791(0.4532,0.74) |

Supplementary Table 12: Distribution of genotypes and genetic model analysis for rs139880713.

|  | Genotype | Case (%) | Control (%) | *P* value | OR (95%CI) |
| --- | --- | --- | --- | --- | --- |
| Allele | C | 20(5.88) | 55(1.53) | 2.01E-08 | 4.024(2.382,6.798) |
|  | T | 320(94.12) | 3541(98.47) |  |  |
| Genotype | CC | 0(0.00) | 0(0.00) | - | - |
|  | CT | 20(11.76) | 55(3.06) | 4.84E-08 | 4.225(2.467,7.238) |
|  | TT | 150(88.24) | 1743(96.94) | - | 1 |
| Recessive model | CC vs. (CT+TT) | 0(0.00)/170(100.00) | 0(0.00)/1798(100.00) | - | - |
| Dominant model | (CC+CT) vs. TT | 20(11.76)/150(88.24) | 55(3.06)/1743(88.24) | 4.84E-08 | 4.225(2.467,7.238) |
| Additive model | CC vs CT vs TT | 0(0.00)/20(11.76)/150(88.24) | 0(0.00)/55(3.06)/1743(96.94) | 3.77E-05 | 3.938(2.052,7.557) |

Supplementary Table 13: Distribution of genotypes and genetic model analysis for rs774409792.

|  | Genotype | Case (%) | Control (%) | *P* value | OR (95%CI) |
| --- | --- | --- | --- | --- | --- |
| Allele | A | 25(7.35) | 82(2.28) | 3.85E-08 | 3.401(2.142,5.400) |
|  | G | 315(92.65) | 3514(97.72) |  |  |
| Genotype | AA | 0(0.00) | 0(0.00) | - | - |
|  | AG | 25(14.71) | 82(4.56) | 6.70E-08 | 3.608(2.235,5.824) |
|  | GG | 145(85.29) | 1716(95.44) | - | 1 |
| Recessive model | AA vs. (AG+GG) | 0(0.00)/170(100.00) | 0(0.00)/1798(100.00) | - | - |
| Dominant model | (AA+AG) vs. GG | 25(14.71)/145(85.29) | 82(4.56)/1716(85.29) | 6.70E-08 | 3.608(2.235,5.824) |
| Additive model | AA vs AG vs GG | 0(0.00)/25(14.71)/145(85.29) | 0(0.00)/82(4.56)/1716(95.44) | 3.26E-05 | 3.264(1.868,5.702) |

Supplementary Table 14: Distribution of genotypes and genetic model analysis for rs117014418.

|  | Genotype | Case (%) | Control (%) | *P* value | OR (95%CI) |
| --- | --- | --- | --- | --- | --- |
| Allele | A | 35(10.29) | 147(4.09) | 1.90E-07 | 2.692(1.829,3.964) |
|  | G | 305(89.71) | 3449(95.91) |  |  |
| Genotype | AA | 2(1.18) | 4(0.22) | 0.1126 | 6.040(1.096,33.273) |
|  | AG | 31(18.24) | 139(7.73) | 4.82E-06 | 2.694(1.759,4.127) |
|  | GG | 137(80.59) | 1655(92.05) | - | 1 |
| Recessive model | AA vs (AG+GG) | 2(1.18)/168(98.82) | 4(0.22)/1794(98.82) | 0.1531 | 5.339(0.971,29.366) |
| Dominant model | (AA+AG) vs GG | 33(19.41)/137(80.59) | 143(7.95)/1655(80.59) | 1.15E-06 | 2.788(1.838,4.229) |
| Additive model | AA vs AG vs GG | 2(1.18)/31(18.24)/137(80.59) | 4(0.22)/139(7.73)/1655(92.05) | 0.3874 | 1.291(0.7232,2.306) |

Supplementary Table 15: Clinical criteria for inclusion.

|  | Subphenotypes of SSNS | |
| --- | --- | --- |
|  | SSNSWR | SDNS/FRNS |
| Basic inclusion criteria:  1.Age at onset of disease in  the study population was 3 months to 18 years.  2.Typical clinical manifestations characteristics of INS.  3. The duration of steroid therapy followed the KDIGO 2012 guidelines. | **Inclusion criteria:**  * Basic inclusion criteria.  Adequate treatment with 2 mg/(kg·d) or 60 mg/(m^2^·d) prednisone results in proteinuria remission after 4 weeks of treatment.  SSNS patients withdraw from steroid treatment and without relapse during the follow-up.  The length of follow-up time should be set to at least 12 months individually. | **Inclusion criteria:**  *Basic inclusion criteria  The following two conditions are met simultaneously:  Two consecutive relapses during the reduction in corticosteroid therapy or within 2 weeks of the discontinuation of corticosteroid therapy.  More than two relapses within a six-month period or relapse for more than 4 of twelve months. |
| **Exclusion criteria:** | 1.Congenital nephrotic syndrome  2.Secondary nephrotic syndrome  3.Positive genetic test associated with podocytopathies  4. Secondary SRNS | |

Supplementary Table 16:Subgroup analysis of the potential SNPs and clinical characteristics.

| SNP | Clinical characteristics | Genotype frequency | | | *P* value | Model analysis | | *P* value | OR (95% CI) |
| --- | --- | --- | --- | --- | --- | --- | --- | --- | --- |
|  | Total relapse times (n) | TT | TC | CC |  | TT+TC | TT |  |  |
| 2:171713702 | 2-3 | 0 | 18 | 86 | 0.022 | 18 | 86 | 0.022 | 4.98 (1.12, 46.13) |
|  | >3 | 0 | 2 | 48 |  | 2 | 48 |  |  |
|  |  | AA | AG | GG |  | AA+AG | GG |  |  |
| rs117014418 | 2-3 | 2 | 12 | 90 | 0.029 | 14 | 90 | 0.043 | 0.40 (0.16, 1.01) |
|  | >3 | 0 | 14 | 36 |  | 14 | 36 |  |  |
|  |  | CC | CT | TT |  | CC+CT | TT |  |  |
| rs139880713 | =2 | 0 | 3 | 78 | 0.004 | 3 | 78 | 0.028 | 3.59 (1.07, 15.62) |
|  | >2 | 0 | 4 | 57 |  | 14 | 59 |  |  |
|  | 2-3 | 0 | 7 | 97 | 0.025 | 7 | 97 | 0.025 | 0.29 (0.09, 0.92) |
|  | >3 | 0 | 10 | 40 |  | 10 | 40 |  |  |
|  | Steroid dose at relapse (mg/kg•d) |  |  |  |  |  |  |  |  |
| 2:171713702 | Less than or equal to 0.75 | 0 | 8 | 26 | 0.034 | 8 | 26 | 0.034 | 3.14 (0.97, 9.87) |
|  | More than 0.75 | 0 | 10 | 103 |  | 10 | 103 |  |  |

**Supplementary Methods: Genotyping and quality control for WES data.**

Genotyping was conducted at the China National Clinical Research Centre (Children's Health and Disease) using the GenCap WES capture kit (MyGenostics, Beijing) and the Illumina NovaSeq platform, and an array of approximately 250,000 SNPs was identified, which was the same array and platform as those of the controls. All SNPs and insertions/deletions (indels) were detected with GATK3.8 software (Broad Institute, Cambridge). SNP-level quality control thresholds, such as call rates lower than 98%, a minimum allele frequency (MAF)<1% and a Hardy–Weinberg equilibrium (HWE) P value<1×10-4, were applied; manual checking of the initially significant variants was conducted. Haploview 4.2 software was used to calculate the LD associated with the filtered SNP data. R-squared correlations (r2s) and standardized disequilibrium coefficients (Ds) were estimated by Haploview, and the means of distances between SNP pairs against r2 values were plotted to visualize the rate of LD decay. Principal component analysis (PCA) was performed by PLINK 2.0 to check for population stratification to identify the subset of patients and controls, and the results were visualized via a principal component (PC) scatter plot. The genomic inflation factor (λGC) and LD score regression, which indicate the level of population stratification in a dataset, were employed for population stratification. The top five PCs of the PCA were used as covariates in the association analysis. Sex discrepancy was assessed by PLINK 2.0.
